# The BOLD signal and neurovascular coupling in autism

**DOI:** 10.1016/j.dcn.2013.07.003

**Published:** 2013-07-12

**Authors:** Clare Reynell, Julia J. Harris

**Affiliations:** Department of Neuroscience, Physiology and Pharmacology, University College London, Gower St, London, WC1E 6BT, UK

**Keywords:** BOLD fMRI, Autism, Blood flow, Neurovascular coupling, Energy, Glutamate

## Abstract

BOLD (blood oxygen level dependent) fMRI (functional magnetic resonance imaging) is commonly used to study differences in neuronal activity between human populations. As the BOLD response is an indirect measure of neuronal activity, meaningful interpretation of differences in BOLD responses between groups relies upon a stable relationship existing between neuronal activity and the BOLD response across these groups. However, this relationship can be altered by changes in neurovascular coupling or energy consumption, which would lead to problems in identifying differences in neuronal activity. In this review, we focus on fMRI studies of people with autism, and comparisons that are made of their BOLD responses with those of control groups. We examine neurophysiological differences in autism that may alter neurovascular coupling or energy use, discuss recent studies that have used fMRI to identify differences between participants with autism and control participants, and explore experimental approaches that could help attribute between-group differences in BOLD signals to either neuronal or neurovascular factors.

## Highlights

• Neurovascular coupling and energy use may be changed in autism.
• The relationship between neural activity and the BOLD signal may be altered in autism.
• Simply comparing the BOLD signal of control and autistic people may not be meaningful.
• Combined techniques will aid the interpretation of group differences in the BOLD signal.

## 1 Introduction

Many important questions in neuroscience and neurology concern differences in brain activity between two or more groups of people. Because fMRI is a powerful and non-invasive tool to examine brain function in humans, it is often employed to read out neuronal activity in such studies. However, for fMRI-based measures of brain activity to be reliably compared between populations, it must be assumed that the relationship between neuronal activity and the BOLD signal is the same in the different groups. This relationship depends on the mechanism by which active neurons alter local blood flow (neurovascular coupling) and on the oxygen use evoked by neuronal activity (Attwell and Iadecola, 2002, Harris et al., 2011).

Several neurophysiological changes have been identified in autism (as discussed below). These changes may alter the signalling from neurons to the vasculature and, in consequence, the relationship between neuronal activity and the BOLD signal. Typically, studies using fMRI to compare control participants and participants with autism attribute observed differences in the BOLD signal to altered neuronal activity, without considering the possibility that neurovascular coupling or oxygen consumption is also altered (Goldberg et al., 2011, Pfeifer et al., 2012, Watanabe et al., 2012). More recently, however, researchers have begun to acknowledge that neurovascular coupling or oxygen use changes must be experimentally ruled out before BOLD differences can be used as evidence for differences of neuronal function between control and autistic groups (Dinstein et al., 2012, Feczko et al., 2012).

In this article, we start by briefly reviewing the physiological basis of the BOLD signal and the neurovascular coupling mechanisms that mediate it. We then look in detail at the neurophysiological changes that are known to occur in autism and how these changes might influence neurovascular coupling and oxygen use, and thus the BOLD response. We then examine the implications that altered neurovascular coupling or oxygen use would have for fMRI studies. By focusing on several representative studies chosen from the literature, we highlight the interpretational problems that can occur when this issue is ignored. Finally, we explore ways in which the scientific community studying autism can address the challenge of separating out neurovascular coupling and neuronal activity effects on the BOLD signal experimentally, as some groups are already beginning to do.

## 2 Neurovascular coupling and the BOLD signal

fMRI is regularly used to study neuronal activity in humans because of its non-invasive nature, which allows it to be used to study a broad range of participants, including patients suffering from pathologies such as autism. It is important to note, however, that the signal produced – the BOLD signal – is not a direct measure of neuronal activity, but instead reflects the operation of several different brain processes associated with neuronal activity, including synaptic transmitter release, the resulting release of signalling molecules that mediate neurovascular coupling and thus increase local blood flow, and (making the BOLD signal smaller) oxygen consumption (Logothetis et al., 2001, Magistretti et al., 1999, Attwell et al., 2010).

The BOLD signal reports changes in the amount of deoxyhaemoglobin in the blood. Deoxyhaemoglobin is paramagnetic, and it decreases MRI signals by making the local magnetic field less homogenous. During neuronal activity, the level of deoxyhaemoglobin initially increases as oxygen use increases, and then decreases as a blood flow increase occurs which over-compensates for the amount of oxygen used. This decrease in the level of deoxyhaemoglobin results in a more homogeneous magnetic field and thus increases the magnetic resonance signal. The size of the BOLD signal is larger when there is a large blood flow increase, but is decreased by the use of oxygen by neurons (for a detailed description, see Logothetis and Wandell, 2004). In what follows we focus simply on the amplitude of the BOLD signal, because although BOLD signals are often analysed in terms of their stimulus-evoked amplitude relative to the ongoing noise, a procedure that requires detailed statistical analysis (Friston, 1995, Friston et al., 2007), even considering just the amplitude of the signal reveals significant concerns about how to compare control and autistic groups of subjects.

When using fMRI to identify differences in neuronal activity between groups, we rely upon the assumption that the BOLD signal represents the same set of processes occurring in these different groups, despite the neurophysiological differences found in pathologies such as autism. Neurophysiological differences may include neuronal activity differences, which is what most researchers are interested in characterising. However, they may also include differences in other processes linked to neuronal activity, such as oxygen consumption or neurovascular coupling. Neurovascular coupling involves several pathways, both neuronal and glial, that lead to the release of vasoactive mediators, such as nitric oxide, prostaglandins and 20-HETE. These alter vessel diameter by promoting either smooth muscle relaxation, which increases vessel diameter, or smooth muscle contraction, which decreases vessel diameter (reviewed by Attwell et al., 2010). Changes in how the release of any of these mediators are controlled by neuronal activity may alter the relationship between neuronal activity and the BOLD signal, potentially obscuring any differences in neuronal activity between the groups studied.

In order to be sure that a BOLD signal difference between groups reflects a neuronal activity difference, it is important that we understand as much as possible about the factors contributing to the BOLD signal. This means considering not only the role of neuronal activity, but also the involvement of other factors, including neurovascular coupling and oxygen consumption, which may differ in certain pathologies.

## 3 Does neurovascular coupling differ in autism?

**Fig. 1:**
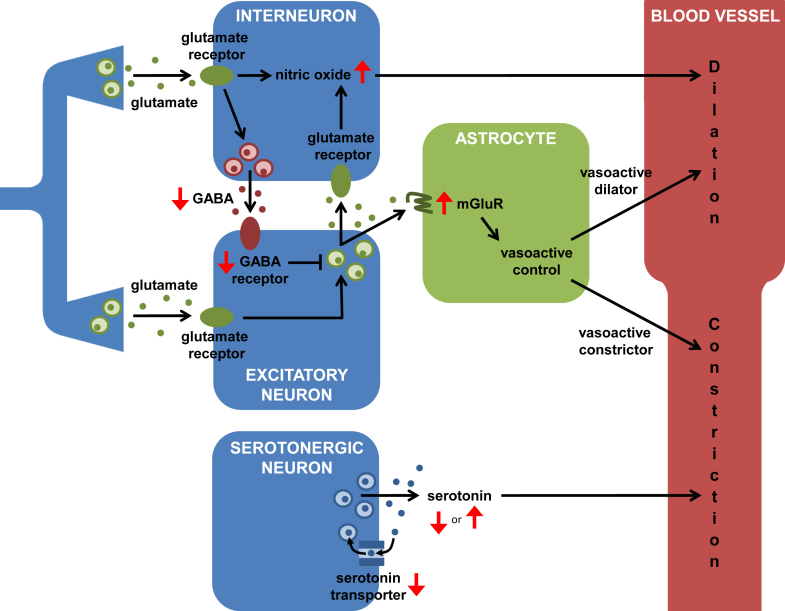
Pathways from interneurons, neurons and astrocytes that regulate blood flow, leading to either dilation (black arrows to upper half of blood vessel) or constriction (black arrows to lower half of blood vessel) of the nearby vasculature. Red arrows indicate how points in these pathways are altered in autism. These changes may affect the relationship between neuronal activity and blood flow response. On the left, excitatory synapses release glutamate onto both interneurons and excitatory neurons. In interneurons, activation of glutamate receptors leads to the production of nitric oxide, a diffusible vasodilatory messenger. It also triggers the release of GABA, which inhibits postsynaptic excitatory neurons, reducing downstream glutamate release. The action of glutamate on astrocytic mGluRs leads to the production of both vasoactive dilators and constrictors (note, recent evidence suggests that mGluRs are only present on astrocytes during infancy; Sun et al., 2013; see text). Serotonin release provides a basal constriction of blood vessels. (For interpretation of the references to colour in this figure legend, the reader is referred to the web version of the article.)

As research into the pathology of autism progresses, we are gaining insight into the neurophysiological abnormalities of the autistic brain (reviewed in Belger et al., 2011), and developing therapeutic strategies to target them (reviewed in Hampson et al., 2012). It is probable that the relationship between neuronal activity and blood flow is affected by some of these abnormalities, or the medications used to treat them, as follows (Fig. 1).

## 4 Abnormalities in neuronal activity

Decreased inhibition is observed in the brains of autistic patients: the expression of the enzymes that synthesise the inhibitory neurotransmitter, GABA, and the expression of the receptors on which GABA acts are both reduced in the autistic brain (Fatemi et al., 2002, Fatemi et al., 2009, Fatemi et al., 2010). This decreased inhibition is a possible therapeutic target for treatment of autism, and several drugs that enhance the action of GABA are currently in phase II clinical trials (divalproex and arbaclofen; clinicaltrials.gov identifier NCT00211757 and NCT00846547, respectively). The effect of decreased inhibition on neuronal activity can be detected by fMRI: because GABA decreases excitability, an autistic brain with impaired GABA function would experience more neuronal excitability and therefore more neuronal firing and more glutamate release, which triggers the release of vasodilators and increases blood flow (Attwell et al., 2010). An increase in BOLD activity would thus be expected, and in this case it would be related to changes in neuronal function.

However, one major issue with this is the potential for nonlinearities in the relationships between inhibition, excitation and blood flow response. For instance, if GABA activity decreases by half, neuronal excitability will not necessarily double – simplistically, it could increase by much more than a factor of two if the depolarising effect of reduced GABA takes most neurons from below to above the threshold for action potential firing, or it could increase by less than a factor of two if the depolarising effect is not sufficient to cross this threshold for most neurons. Similarly, if neuronal excitability doubles, the blood flow response will not necessarily double – it could increase by very little if blood vessels are already near-maximally dilated, which may be the case much of the time in brains where reduced GABA function increases the level of resting excitability. A further complication comes from the recent suggestion that GABA released from interneurons may act on glial cells or directly on microvessels to increase blood flow (Kocharyn et al., 2008). A reduced action of this pathway in autistic patients would lead to a decreased BOLD signal, unrelated to neuronal excitability.

How the BOLD signal is affected overall by the change of GABAergic inhibition in autism will depend, therefore, on the relative magnitude of these opposing effects of GABA on blood flow, which may vary between brain regions.

## 5 Abnormalities in vasoactive mediators

Other neurophysiological changes observed in autism include alterations in the signalling pathways mediating neurovascular coupling (for a review of neurovascular coupling pathways, see Attwell et al., 2010). For instance, there is a possible role for nitric oxide – a vasodilator released by neurons in response to activity – in the pathogenesis of autism. A study using an animal model of autism, involving prenatal human influenza viral infection, found region- and time-dependent changes in the expression of nNOS, the neuronal enzyme which produces nitric oxide. Expression of nNOS was increased in rostral brain areas at adolescence but eventually down-regulated in adulthood (Fatemi et al., 2000). Support for increased NO activity during development in autism has also been found by assessment of NO production. Expression of adrenomedullin, which stimulates NO release, and generation of nitrite, a metabolic product of NO, are increased in children with autism (Zoroğlu et al., 2003). These findings suggest that, during development, more nitric oxide will be released in response to the same amount of activity, which would lead to an increased haemodynamic response (and BOLD signal) in autistic versus control groups.

The expression of a specific subtype of metabotropic glutamate receptor, mGluR5, is also increased in children – but not adults – with autism (Fatemi et al., 2011, Fatemi and Folsom, 2011). Activation of these receptors on astrocytes by glutamate released during synaptic transmission leads to the production and release of several vasoactive messengers, which can either increase or decrease blood flow (Attwell et al., 2010). Interestingly, recent evidence suggests that mGluR5 is only present on astrocytes during infancy; in adulthood, mGluR5 expression stays high in neurons but is diminished in astrocytes (Sun et al., 2013). Thus, it is possible that the increased mGluR5 in early autism is associated with excess expression in astrocytes, which would imply an abnormal relationship between synaptic activity and haemodynamic response in children – but not necessarily adults – with autism. Alternatively, the increased mGluR5 expression in children with autism could have nothing to do with astrocytes – the Western blot analyses carried out on post-mortem tissue did not have cell-type resolution (Fatemi et al., 2011, Fatemi and Folsom, 2011). Determining whether the upregulation of mGluR5 in children with autism is associated with neuronal or astrocytic expression will be key to categorising this change as a relevant or confounding factor in the interpretation of BOLD signal differences.

Finally, global serotonin synthesis is abnormally low in children with autism but, in adolescence, it increases gradually to 1.5 times the level in adult controls (Chugani et al., 1999a). In children with autism, the action of the presynaptic transporter responsible for active serotonin reuptake after synaptic signalling is also reduced (Makkonen et al., 2008). The authors suggest that this is because fewer serotonergic terminals, and therefore transporters themselves, are present in children with autism. If the reduction in serotonin synthesis and release is matched or exceeded by the reduction in serotonin reuptake, this would be in line with the general view that the action of serotonin is decreased in early autism. On the other hand, if the number of transporters is reduced beyond the reduction in serotonin release, this would tend to increase the action of synaptically released serotonin. Because serotonin is thought to produce a basal constriction of blood vessels (Cohen et al., 1997), either a decrease or an increase in serotonergic activity could change vessel tone, and thus alter the vessel response to the vasodilators released by a given amount of neuronal activity (Blanco et al., 2008). For example, in low levels of serotonin, a vessel may already be close to its maximal diameter and thus be less able to dilate in response to neuronal activity, resulting in a smaller BOLD response. Conversely, in high levels of serotonin, an highly constricted vessel could allow a larger dilation in response to the same amount of activity, leading to a larger BOLD response. Alternatively, increased vessel tone could make it more difficult for the vessel to dilate (if a larger vasodilatory signal were needed to overcome the constriction), leading to a smaller BOLD response. More research is required before we can predict exactly how altered basal tone will affect activity-evoked blood flow responses.

## 6 Abnormalities in energy use

Although some evidence suggests that there is no change in metabolism (DeVolder et al., 1987, Herold et al., 1988, Kaya et al., 2002), several studies find metabolic alterations in autism (Rumsey et al., 1985, Chugani et al., 1999b, Weiss et al., 2007, Weiss et al., 2008, Weiss et al., 2009, Weiss et al., 2012). For instance, Rumsey et al. (1985) used positron emission tomography to show that glucose utilisation was higher in adult males with autism than in controls. In an animal model of autism, the Eker rat, basal oxygen consumption was found to be as much as 50% higher than that of control rats (Weiss et al., 2007).

This increase in glucose and oxygen use could indicate a greater need for energy in the autistic group. In line with this is the suggestion that the increased oxygen use and cerebral blood flow in Eker rats is due to a reduced basal activity of GABA_A_ receptors (Weiss et al., 2008), which would lead to an overall increase in neuronal activity, and therefore a greater energy demand. However, the increased oxygen use in these rats was not associated with an increase in NMDA or AMPA receptor activation (Weiss et al., 2007, Weiss et al., 2009, Weiss et al., 2012), suggesting that there may be an increase in the activity of non-glutamatergic neurons.

Alternatively, the autistic brain may require more metabolic substrate to produce the same amount of energy. This is supported by increasing evidence that the function of the mitochondrial electron transport chain (ETC) is impaired in autism. Compared to controls, a reduction in the expression of ETC genes (Anitha et al., 2012) and proteins (Chauhan et al., 2011) was seen in the post-mortem brain tissue of patients with autism. Thus, oxidative phosphorylation may not be as effective in the autistic brain, perhaps requiring more oxygen and/or glucose to produce the same amount of ATP. Since the amount of energy needed to restore ionic concentrations after neuronal signalling is fixed by the amount of sodium ion entry occurring (Attwell and Laughlin, 2001), this would imply a larger oxygen requirement to support the same level of neuronal activity. If this excess oxygen were obtained by increased oxygen extraction from the blood, then this would lead to a smaller BOLD response. However, in the mouse model of autism, oxygen extraction is unchanged while cerebral blood flow is increased (Weiss et al., 2007, Weiss et al., 2008, Weiss et al., 2012), suggesting that the excess oxygen is obtained by increased vascular supply. This would lead to a larger BOLD response to the same amount of neuronal activity in people with autism.

## 7 Medication-related effects on neurovascular coupling

The medication used to treat autism may also affect the relationship between neuronal activity and the BOLD response. For example, serotonin reuptake inhibitors (SSRIs), a medication commonly used by autistic patients, increase extracellular serotonin levels in the brain. They may, therefore, increase the tone of vessels – perhaps beyond normal levels – and thus alter the blood flow response evoked by neuronal activity (see above). A recent study by Feczko et al. (2012) addressed this question, finding no significant difference in the BOLD response to a visuomotor task presented to non-medicated participants and those taking SSRIs, in the brain areas they examined. However, this does not rule out SSRI-induced neurovascular coupling changes in the brain circuits that underpin the social behaviours more closely associated with the autistic phenotype.

## 8 Potential impact of neurovascular coupling changes in autism on fMRI studies

Having highlighted some of the neurophysiological differences found in autism that may alter the relationship between neuronal activity and the BOLD response in this condition, we will now consider some fMRI studies in which the results may be affected by these differences.

Goldberg et al. (2011) asked children with or without high functioning autism spectrum disorder (ASD) to carry out an error monitoring task. After a trial in which an error was made, the BOLD signal in the anterior medial prefrontal cortex and the superior temporal gyrus was found to increase in ASD participants compared to control participants. This increase in the BOLD signal was interpreted as increased neuronal activity in these regions. Because the behavioural performance on the task was the same for both groups, the assumed increase in neuronal activity was suggested to indicate a “greater attention towards the internally-driven emotional state associated with making an error” in children with ASD. But the authors did not take any steps to rule out the possibility that the difference between these groups may be neurovascular in nature, and nothing to do with neuronal activity. For instance, the increased nitric oxide level found in children with autism (Zoroğlu et al., 2003) could lead to a larger vascular response to activation of the same number of neurons (see above). This would cause the BOLD response to appear larger in children with autism, despite no difference in neuronal activity.

Watanabe et al. (2012) found that the BOLD response to a social judgement task was decreased in participants with ASD compared to control participants. The degree of BOLD signal reduction in the two regions most active in control participants during this task (the anterior cingulate cortex/ventromedial prefrontal cortex and the dorsal medial prefrontal cortex) predicted the severity of communication deficit in the ASD group. This result might suggest that some physiological abnormality in these regions is associated with the behavioural abnormality in social judgement. However, the idea that the physiological abnormality is reduced neuronal activity can only be an assumption until other possibilities are ruled out. For example, the decreased serotonin levels seen in autistic patients (Chugani et al., 1999a) may restrict the amplitude of the vascular and thus the BOLD response (see above). The authors point out that the reduction in the BOLD response in autistic participants is not global, as task-evoked activity in one area, the amygdala, was found to increase. However, neurovascular coupling mechanisms can be pathway-specific (Enager et al., 2009) and, therefore, task-specific. It is thus possible that changes in neurovascular coupling are responsible for either the decreased or increased BOLD responses found by Watanabe et al. (2012).

Pfeifer et al. (2012) compared the BOLD signal when participants were asked to make an appraisal of themselves or of others. In typically developing children and adolescents, two main brain regions – the medial prefrontal cortex (mPFC) and the middle cingulate cortex (MCC) – showed an increased BOLD response during self-appraisal compared to appraisal of others. Age-matched participants with ASD lacked any task-related pattern in the mPFC, and showed the opposite pattern in the MCC (increased BOLD signal when appraising others compared to self). However, with increasing age in the ASD group, the BOLD signal in the mPFC associated with the differentiation of the self from others was found to increase. This trend suggests that this brain region is increasingly relied upon as the social importance of the self-other distinction is learned. However, the interpretation of age-related BOLD changes comes with its own set of issues, as the neurovasculature matures (Harris et al., 2011), perhaps differently in autistic and typically developing populations.

## 9 Experimentally excluding neurovascular coupling differences

Since most researchers are interested in the neuronal changes associated with autism, most changes in fMRI response are simply assumed to be due to altered neuronal processing rather than altered neurovascular coupling or oxygen use. None of the studies above considered neurovascular abnormalities in autism, which, if present could directly affect the BOLD signal even in the absence of changes in neuronal activity. The mislabelling of neurovascular changes as neuronal activity changes could be misleading. The best way to avoid this is with an experimental design that can distinguish a neurovascular coupling (or oxygen use) change from a neuronal activity change. A few labs have begun to design experiments with this goal in mind.

Feczko et al. (2012) asked children with and without ASD to press a button at the onset and offset of a visually presented flickering chequerboard, and compared BOLD responses in the two groups during this task in 19 different brain regions. The same paradigm has previously been employed to compare BOLD responses across age groups (Kang et al., 2003). This approach assumes that the neuronal activity mediating a simple, low-level task is not affected by autism (or age). This assumption may not be valid, since altered visual processing has been reported in the retina and striate cortex in autism (Ritvo et al., 1988, Simmons et al., 2009, Neumann et al., 2011; but see Koh et al., 2010). But if it is a correct assumption, then identical BOLD responses in the two groups would presumably imply that neurovascular coupling is the same in the two groups.

Since Feczko et al. found no significant difference in the visual task-evoked BOLD response between ASD and control children, they argued that neurovascular coupling in the two groups must be the same, ideally throughout the brain. This is the most parsimonious argument, but the authors do acknowledge the small possibility that a confounding difference in neurovascular coupling that has equal and opposite effects to a difference in neuronal activity between the groups could lead to the same overall BOLD signal. For example, a smaller amount of neuronal activity in autism may be compensated for by a stronger neurovascular signalling pathway, or vice versa. In this case, a similar blood flow response would be produced in the different groups by differing levels of neuronal activity.

Even if one accepts the argument that a similar BOLD response for a low level visual task implies similar neurovascular coupling, as noted by the authors, it can only be made for this particular visual task and need not generalise to other tasks. As mentioned above, this is because different tasks activate different brain regions, different neuronal pathways and different neurotransmitter systems, which vary in their neurovascular coupling mechanisms (Sloan et al., 2010, Devonshire et al., 2012). Indeed, even within the same brain region, neuronal activity evoked by different inputs can generate different BOLD responses (Enager et al., 2009).

Such input- or task-specific neurovascular coupling makes it difficult for results based on one task alone to be extrapolated usefully to general claims about neurovascular coupling between groups – it is always a possibility that the chosen task happens to be one in which neuronal responses and neurovascular coupling are not altered between groups, while for tasks employing different brain regions and pathways there may indeed be differences of neurovascular coupling between the groups. For example, in Feczko et al.’s case, it is possible that low level visual areas do not have their neurovascular coupling altered by the neuronal changes associated with autism, while brain areas dealing with social interactions do have their neurovascular coupling altered. Nevertheless, this study represents the first attempt to experimentally rule out neurovascular coupling confounds in the interpretation of fMRI data, and paves the way for paradigms which address this issue directly.

Dinstein et al. (2012) used a different approach to exclude the possibility that an increase in the variability of neurovascular coupling in autism might underpin the greater fMRI response variability that they observed. Specifically, they found that, although mean evoked responses to several stimuli (visual, auditory, and somatosensory) did not differ between participants with and without autism, there was a significant increase in trial-by-trial variability in participants with autism. They compared these task-evoked responses in visual, auditory or somatosensory regions (“local evoked activity”) with the fMRI fluctuations that were common to the entire cortex in the absence of stimulus-evoked responses (“global ongoing activity”), which did not show increased variability in participants with autism. Because the authors started with the assumption that a change in neurovascular coupling would affect “evoked responses and ongoing activity in a similar manner”, they argued that neurovascular coupling changes could therefore not be the source of the increased variability in the local task-evoked BOLD signals. The conclusion followed that task-related neuronal activity is abnormally unreliable in autism.

This line of reasoning on its own is weak, because there is no reason why a neurovascular coupling change could not selectively alter task-evoked signalling to the vasculature without affecting resting state blood flow. This is obvious when one considers that the majority of neurovascular coupling changes are likely to occur at points along the pathway from synaptic transmission to vessel dilation/constriction, and may therefore only be revealed during task-evoked synaptic activity.

Nevertheless, Dinstein et al.’s conclusions are supported by EEG data showing that visual task-evoked response variability is significantly greater in participants with ASD (Milne, 2011). The combination of fMRI with a direct measure of neuronal activity, such as EEG, is one of the most hopeful approaches for experimentally dissecting out the relative contributions of altered neuronal activity and neurovascular coupling to differences in the BOLD signal. For instance, an increased BOLD response echoed by an increased EEG response to the same task is a very good indication that the BOLD increase reflects increased neuronal activity. The fMRI data can then be used to gain a clearer spatial picture of the change. If, however, BOLD and EEG data contradict one another, this would indicate that other factors – perhaps a change in neurovascular coupling or energy use – are contributing to the BOLD signal difference. In this way, performing EEG alongside fMRI can guide the identification of the neuronal differences between groups.

## 10 Conclusion

Autism is a brain disorder, with its diverse behavioural phenotypes rooted in abnormalities of neuronal circuitry and function, and we do not wish to suggest that fMRI cannot reveal these neuronal differences. The aim of this review is to dispel the idea that neurovascular coupling can be lumped in with easily corrected for non-neuronal confounds such as head motion or breathing artefacts. On the contrary, neurovascular coupling changes can have profound, complex and region- and task-specific effects on cerebral blood flow and therefore the BOLD signal.

Autism is associated with many neurophysiological changes, and there are just as many reasons why an abnormal relationship between neuronal activity and the BOLD signal might therefore be expected. Being aware of these potential neurovascular coupling and energy use differences will be critical to the field's ability to dissect out the neuronal changes that underpin behavioural differences. Eventually, detailed knowledge of the physiology of the BOLD signal (Attwell and Iadecola, 2002) will allow more confident interpretation of BOLD response differences between participants. The most promising way to achieve this is by using a combination of imaging and physiological research in animals (Devonshire et al., 2012, Schulz et al., 2012). Meanwhile, an experimental approach – based around carefully chosen control tasks or a combination of fMRI and EEG (or, indeed, any method that reports the electrical activity of neurons, such as MEG) – is the best strategy to distinguish the contributions of neurovascular coupling and of neuronal activity to differences in BOLD signals between groups.

## Conflict of interest

The authors declare no conflict of interest.
